# The high bone mass phenotype is characterised by a combined cortical and trabecular bone phenotype: Findings from a pQCT case–control study^☆^

**DOI:** 10.1016/j.bone.2012.10.021

**Published:** 2013-01

**Authors:** Celia L. Gregson, Adrian Sayers, Victor Lazar, Sue Steel, Elaine M. Dennison, Cyrus Cooper, George Davey Smith, Jörn Rittweger, Jon H. Tobias

**Affiliations:** a Musculoskeletal Research Unit, University of Bristol, Avon Orthopaedic Centre, Southmead Hospital, Bristol BS10 5NB, UK; b MRC Lifecourse Epidemiology Unit, University of Southampton, Southampton General Hospital, Southampton SO16 6YD, UK; c School of Social and Community Based Medicine, University of Bristol, Canynge Hall, 39 Whately Road, Bristol BS8 2PS, UK; d Centre for Magnetic Resonance Investigations, Hull and East Yorkshire NHS Trust, Anlaby Road, Hull HU3 2JZ, UK; e Hull and East Yorkshire NHS Trust, Anlaby Road, Hull HU3 2JZ, UK; f MRC Centre for Causal Analyses in Translational Epidemiology (CAiTE). School of Social and Community Based Medicine, University of Bristol, Oakfield House, Oakfield Grove, Bristol BS8 2BN, UK; g Institute for Biomedical Research into Human Movement and Health, Manchester Metropolitan University, All Saints Building, All Saints Manchester M15 6BH, UK; h Institute of Aerospace Medicine, German Aerospace Center, Cologne, Germany

**Keywords:** HBM, high bone mass, NHS, National Health Service, pQCT, peripheral quantitative computed tomography, OA, osteoarthritis, L1, 1st lumbar vertebra, cBMD, cortical bone mineral density, tBMD, trabecular bone mineral density, TBA, total bone area, CBA, cortical bone area, SSI, strength strain index, SD, standard deviation, PVE, partial volume effect, High bone mass, pQCT, Cortical, Trabecular, Age, BMD

## Abstract

High bone mass (HBM), detected in 0.2% of DXA scans, is characterised by a mild skeletal dysplasia largely unexplained by known genetic mutations. We conducted the first systematic assessment of the skeletal phenotype in unexplained HBM using pQCT in our unique HBM population identified from screening routine UK NHS DXA scans.

pQCT measurements from the mid and distal tibia and radius in 98 HBM cases were compared with (i) 65 family controls (constituting unaffected relatives and spouses), and (ii) 692 general population controls.

HBM cases had substantially greater trabecular density at the distal tibia (340 [320, 359] mg/cm^3^), compared to both family (294 [276, 312]) and population controls (290 [281, 299]) (*p* < 0.001 for both, adjusted for age, gender, weight, height, alcohol, smoking, malignancy, menopause, steroid and estrogen replacement use). Similar results were obtained at the distal radius. Greater cortical bone mineral density (cBMD) was observed in HBM cases, both at the midtibia and radius (adjusted *p* < 0.001). Total bone area (TBA) was higher in HBM cases, at the distal and mid tibia and radius (adjusted *p* < 0.05 versus family controls), suggesting greater periosteal apposition. Cortical thickness was increased at the mid tibia and radius (adjusted *p* < 0.001), implying reduced endosteal expansion. Together, these changes resulted in greater predicted cortical strength (strength strain index [SSI]) in both tibia and radius (*p* < 0.001). We then examined relationships with age; tibial cBMD remained constant with increasing age amongst HBM cases (adjusted β − 0.01 [− 0.02, 0.01], *p* = 0.41), but declined in family controls (− 0.05 [− 0.03, − 0.07], *p* < 0.001) interaction *p* = 0.002; age-related changes in tibial trabecular BMD, CBA and SSI were also divergent. In contrast, at the radius HBM cases and controls showed parallel age-related declines in cBMD and trabecular BMD.

HBM is characterised by increased trabecular BMD and by alterations in cortical bone density and structure, leading to substantial increments in predicted cortical bone strength. In contrast to the radius, neither trabecular nor cortical BMD declined with age in the tibia of HBM cases, suggesting attenuation of age-related bone loss in weight-bearing limbs contributes to the observed bone phenotype.

## Highlights

► High Bone Mass (HBM) is characterised by increased bone size, cortical BMD, cortical thickness and increased strength strain index. ► In HBM, trabecular density is also increased at both the distal radius and tibia. ► Tibial cortical and trabecular BMD decline with age in controls, but not in HBM implying attenuation of age-related loss. ► Similar differences in age-related bone loss are not seen at the radius, suggesting a possible interaction with weight-bearing.

## Introduction

High bone mass (HBM) is a sporadic finding of generalised raised bone mineral density (BMD) on dual-energy X-ray absorptiometry (DXA) scanning, and when defined as such has a prevalence of 0.2% amongst a UK DXA-scanned population [1]. In a family of HBM cases due to activating *low-density lipoprotein receptor-related protein 5* (*LRP5*) gene mutations, which enhance osteoblast activity, radiographs have shown widened long bones and cortices [2]. More recently high resolution peripheral quantitative computed tomography (HRpQCT) scanning of 19 individuals, from 4 families, with HBM caused by a *T253I LRP5* mutation has identified increased cortical and trabecular BMD at the distal tibia [3]. However, much HBM is not explained by established *LRP5* mutations, and detailed characterisation of bone structure in a large population of individuals with this unexplained HBM has yet to be described. Within such a HBM population it is not known whether HBM is associated with features of enhanced bone modelling (e.g. increased periosteal expansion) or reduced bone remodelling (e.g. reduced endosteal expansion), increased trabecular or cortical bone mass, nor whether the phenotype results from enhanced peak bone mass accrual or reduced age-related bone loss. Recent studies of heterogeneous populations with low bone mass have provided important insights into the pathogenesis of osteoporosis [4]. Thus examining individuals with excess bone mass, identified as a population extreme, is anticipated to be equally informative.

We have collected a unique HBM population; having screened 335,115 historical DXA scans across 13 UK National Health Service (NHS) centres for BMD Z/T-scores ≥+ 4. We have previously described the associated clinical characteristics suggestive of a mild skeletal dysplasia in those with unexplained HBM [1]. We recruited a contemporaneous family control population, comprising unaffected relatives and spouses [1]. However, family controls can be expected to be more similar to cases, due to shared environmental and inherited factors, than unrelated controls sampled from the general population. Hence, in exploring the phenotype of our HBM cases, additional comparison is needed with unrelated general population controls, with the expectation that the characteristics of family controls lie between those of HBM cases and general population controls.

Peripheral quantitative computed tomography (pQCT) is a low radiation dose research tool enabling measurement of key components of bone geometry which conventional DXA is unable to assess. In the present study, we performed the first systematic evaluation of the skeletal phenotype of HBM individuals sampled from the UK DXA population, assessed using pQCT. In particular, we aimed to establish to what extent alterations in cortical and/or trabecular bone contribute to the increased bone mass observed in HBM, to characterise changes in bone structure underlying these findings, and to determine to what extent altered age-related bone loss contributes to the observed phenotype.

## Methods

### Participant recruitment

#### High bone mass cases and family controls

The HBM study is a UK based multi-centred observational study of adults with unexplained HBM. This pQCT study was limited to our largest study centre, where 196 cases of unexplained HBM were identified by screening a NHS GE Lunar DXA database (*n* = 105,333) (Hull Royal Infirmary). Full details of DXA database screening and participant recruitment have previously been reported [1]. In brief, HBM was defined as (a) L1 Z-score of ≥+ 3.2 plus total hip Z-score of ≥+ 1.2 or (b) total hip Z-score ≥+ 3.2 plus L1 Z-score of ≥+ 1.2. Cases with significant osteoarthritis (OA) and/or other causes of raised BMD were excluded (e.g. Paget's disease, malignancy, artefacts, etc.). L1 was used as it was not associated with the presence of OA, reflecting the recognized pattern of progressive OA changes seen in descending sequential lumbar vertebrae [5]. Index cases were asked to pass on study invitations to their first-degree relatives and spouse/partner(s). Relatives/spouses with HBM were in turn asked to pass on study invitations to their first-degree relatives and spouses. First-degree relatives and spouses were recruited; in these individuals, HBM status was defined as the sum of L1 plus total hip Z-scores of ≥+ 3.2. Family controls comprised unaffected relatives as defined in this manner, and spouses. Spouses were recruited to increase sample size, reduce residual confounding from unmeasured environmental factors shared with HBM cases and who, as a function of their genetic independence, would be unlikely to share common polygenic influences over BMD. Recruitment ran from September 2008 until April 2010. All participants were clinically assessed by one doctor using a standardised structured history and examination questionnaire, after which DXA scans were performed for relatives and spouses, using local GE Lunar Inc. Madison, WI, USA) DXA systems applying manufacturer's standard scan and positioning protocols, and weight and routine height measurements were recorded. Body mass index (BMI) was calculated as weight (kilograms)/height (metres)^2^. Current and historical physical activity data were collected from HBM cases and family controls by questionnaire (including the validated international physical activity questionnaire [IPAQ] [6–9]). Participants were excluded if under 18 years of age, pregnant or unable to provide written informed consent for any reason.

#### Population controls

The Hertfordshire Cohort Study is a population based cohort study tracing 42,974 men and women born in Hertfordshire during 1931–1939 and still living there during the period 1998–2003. Individuals were traced using the NHS central registry at Southport and the Hertfordshire Family Health Service Association. Full details of the study design have previously been reported [10]. A planned subsample of 6099 individuals were invited to participate in a clinical study and 3225 (53%) men and women aged 60–75 years were recruited and completed home interviews [10]. In 2004 and 2005 a subgroup (from East Hertfordshire) were followed up and 322 men (65%) and 321 women (69%) re-attended, completed lifestyle questionnaires which included questions concerning medical history including fractures, smoking and alcohol consumption. Height was measured to the nearest 0.1 cm using a Harpenden pocket stadiometer and weight to the nearest 0.1 kg using floor scales, at the time of pQCT assessment [11].

### pQCT methods

pQCT scans were performed at the distal and mid-shaft of the tibia (4 and 66% from the distal endplate) in the non-dominant lower limb using a Stratec XCT2000L (Stratec Medizintechnik, Pforzheim, Germany); voxel size 0.5 mm, CT speed 30 mm/s, XCT software version 5.50d. A reference line at the distal endplate was determined from initial frontal scout view. Cortical bone was defined using a threshold above 650 mg/cm^3^ (optimal for bone geometry [12]). Trabecular bone was identified by elimination of cortical bone and therefore trabecular bone mineral density (tBMD) was defined as a density < 650 mg/cm^3^. Cortical thickness, periosteal circumference and endosteal circumference were derived using a circular ring model. Further cortical parameters were measured: cortical bone mineral density (cBMD), total bone area (TBA) (i.e. total bone cross-section, reflecting periosteal expansion), cortical bone area (CBA) (reflecting a combination of periosteal and endosteal expansion) and CBA/TBA (%). Strength strain index (SSI) was calculated according to Stratec's user manual (SSI = SM*(cBMD[mg/cm^3^]/1200[mg/cm^3^]), where 1200 mg/cm^3^ represents the normal physiological density of bone (stated by Stratec) and SM (Section Modulus) = CSMI/periosteal radius, where CSMI (cross-sectional moment of inertia [cm^4^]) = Π(periosteal radius^4^ − endosteal radius^4^)/4) [13]. Twenty population controls were scanned twice on the same day after repositioning and measurement precision (CV) was typically between 1 and 3% [11]. Stratec pQCT machines were calibrated using a COMAC phantom; mean (SD) difference between scanners was 1.18 (0.82) %. Data acquisition and analysis methods were the same for all cases and controls.

pQCT scans were also performed at the distal and mid-shaft of the radius (4 and 60% from the distal endplate) in the non-dominant upper limb. The 60% site was not scanned in population controls, so comparisons could not be made.

### Ethics

Written informed consent was collected for all participants in line with the Declaration of Helsinki [14]. This research was approved by the Bath Multi-centre Research Ethics Committee (REC), the North and East Yorkshire and Northern Lincolnshire NHS Local REC and the East and North Hertfordshire Ethical Committees.

### Statistical methods

Descriptive statistics are presented as mean (standard deviation [SD]) for continuous and count (percentages) for categorical data. Linear regression was used to analyse continuous pQCT variables, which were normally distributed. A random effects model was used in HBM case-family control analyses to allow for the lack of statistical independence due to within-family clustering of environmental factors and shared genotypes. Age, gender and menopausal status in women were considered *a priori* confounders of the associations between HBM status and all pQCT geometric parameters. Further confounders included weight, height, limb length, smoking status, alcohol intake, physical activity, previous or current use of steroids, estrogen replacement, or experience of malignancy (which also acted as a proxy for use of aromatase inhibitors for breast cancer and anti-androgens for prostate cancer). Adjusted means and mean differences with 95% confidence interval [CI] are presented for two sets of analyses: (i) HBM cases vs. family controls, (ii) HBM cases vs. population controls. Further analyses of continuous variables by age, stratified by case–control status, are presented as adjusted β coefficients and 95% CIs for standardized outcomes. Data were analysed using Stata release 11 statistical software (StataCorp, TX, USA).

## Results

In total 98 HBM cases (71 index cases and 27 affected relatives), 65 family controls (43 unaffected relatives and 22 unaffected spouses) and 692 population controls were assessed. HBM cases (age range 26–87 years) were younger than population controls (range 65–74 years), but older than family controls (range 19–88 years) (Table 1). HBM cases were heavier with greater BMI than both control groups. A higher proportion of HBM cases were female than in the control groups, and although population controls were almost all postmenopausal, HBM cases had more experience of estrogen replacement therapy. Age at menarche was similar between HBM cases and family controls (mean [SD] 12.8 [1.6] and 12.6 [1.5] years respectively, *p* = 0.869). HBM cases were more likely to report a history of cancer and steroid use. No participants gave a history of hepatitis C or excess fluoride ingestion. All study participants were of white European origin. No consanguinity was reported.

### Tibia

#### Bone size

**Fig. 1:**
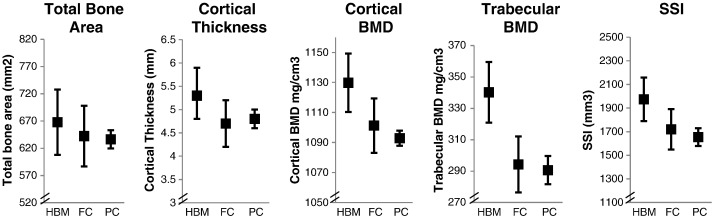
Fully adjusted distal and mid-tibia pQCT measures in high bone mass cases compared with family and population controls. BMD: bone mineral density, SSI: strength strain index, HBM: high bone mass cases, FC: family controls, PC: population controls. Means and 95% CI shown adjusted for age, weight and height, alcohol consumption, smoking status, malignancy and steroid use, and menopausal status and estrogen replacement use in women. Mid-tibia total bone area shown. FC compared with HBM, PC compared with HBM, *p* < 0.001 for all except total bone area for FC where *p* = 0.05. PC radial measures were not available hence presented results limited to the tibia.

In unadjusted analyses, HBM cases had substantially greater TBA at the distal tibia (4% site) than both family and population controls (Table 2). Similar results were obtained after adjustment for confounding factors (age, gender, weight and height, alcohol consumption, smoking status, malignancy and steroid and estrogen replacement use), with a mean difference of just over 2 cm^2^, between HBM cases and both control groups (equivalent to a 19% increase above that of both family and population controls) (Table 3, Fig. 1). At the mid-tibia (66% site), after similar adjustment TBA was also greater in HBM cases compared with both control groups, although this difference was smaller in proportion to those changes observed distally; mid-tibial TBA in HBM cases was approximately 4% and 8% larger compared with family and population controls respectively (Table 3, Fig. 1). Consistent with these increases in TBA, mid-tibia periosteal circumference was also increased in HBM cases compared with family controls (adjusted mean difference 1.72 [95%CI − 0.06, 3.49] mm, *p* = 0.058) and population controls (3.80 [2.59, 5.00] mm, *p* < 0.001).

#### Cortical dimensions

Mid-tibial cortices were thicker in HBM, in unadjusted and adjusted analyses, as compared with both family and population controls (Tables 2 and 3). After adjustment HBM cases had on average 0.5 mm thicker cortices compared with family and population controls respectively (Table 3, Fig. 1). Furthermore, at the mid-tibia, CBA and CBA/TBA were also greater in HBM cases compared with both control groups, suggesting a greater proportion of the cross-section of bone was cortical. Although cortical thickness measured distally can be unreliable, before adjustment HBM cases appeared to have increased cortical thickness compared with population controls (Table 2). After adjustment HBM cases had on average 37% and 112% thicker cortices compared with family and population controls respectively (Table 3).

#### Bone density and strength

Both trabecular and cortical BMD, measured at the distal and mid-tibia respectively, were greater in HBM cases than family controls, and greater still when compared with population controls, both before and after adjustment for confounding factors (Tables 2 and 3, Fig. 1). After adjustment for confounding factors, SSI at the mid-tibia was substantially higher in HBM cases compared with both control groups (as were CSMI and SM, data not shown).

### Radius

#### Bone size

Consistent with observations at the tibia, TBA at the distal radius was also greater (by approximately 20% after adjustment for confounders detailed above) in HBM cases compared with both control groups (supplementary Tables 1s and 2s). However, differences in mid-radial TBA between HBM cases and family controls were only apparent after adjustment, when the difference was approximately 5%.

#### Cortical dimensions

Similarly, at the mid-radius, only after adjustment did HBM cases have thicker cortices than family controls (e.g. 3 mm mean difference), and of a lesser magnitude to that observed in the lower limb. At the mid-radius, both CBA and CBA/TBA were higher in HBM cases; however, again these differences were not as overt as those seen in the lower limb. Bearing in mind pQCT resolution limitations, after adjustment distal cortical thickness was also greater in HBM cases compared with both family and population controls (supplementary Table 2s).

#### Bone density and strength

Findings from the radius were consistent with those in the tibia. Both trabecular and cortical BMD, measured at the distal and mid-radius respectively, were greater in HBM cases compared with controls, both before and after adjustment for confounding factors, although differences in radial tBMD were smaller than those seen in the tibia (supplementary Tables 1s and 2s). Only after adjustment was a difference observed in terms of greater radial SSI amongst HBM cases compared with family controls.

### Further analyses

In general, gender stratified analyses revealed similar differences between HBM cases and control groups in males and females (Table 4, unadjusted results shown in supplementary Table 3s); no evidence was detected to support a gender interaction. Results comparing HBM cases and family controls were not materially affected by adjustment for limb length rather than height, or by further adjustment for questionnaire-assessed physical activity (data not shown).

#### Relationships between pQCT parameters and age: Tibia

**Fig. 2:**
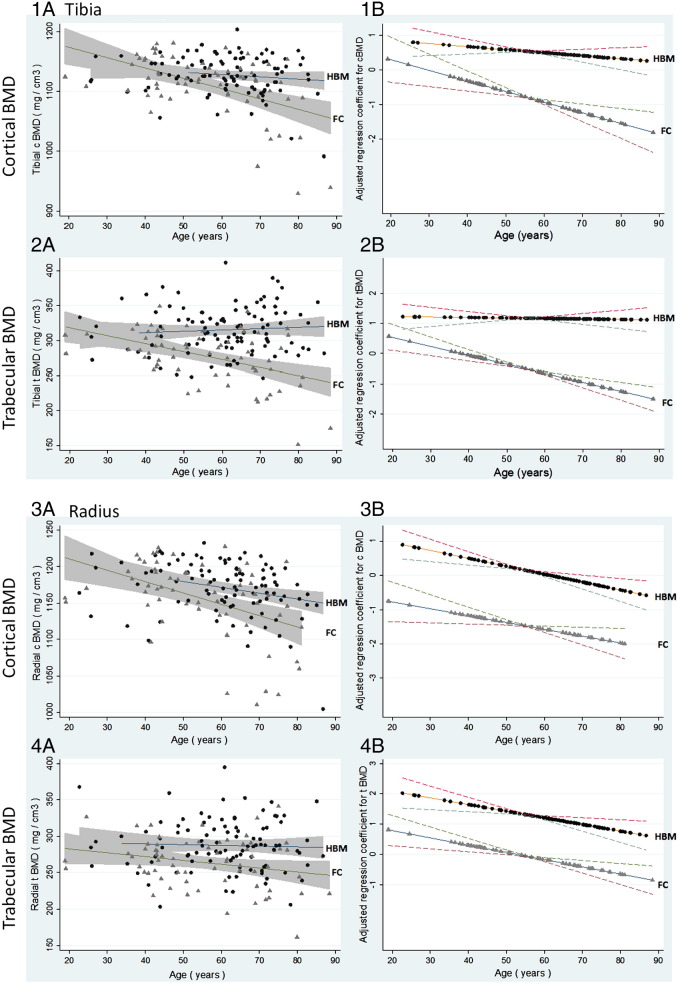
Unadjusted and fully adjusted regressions for changes in cortical and trabecular BMD by age in HBM cases and family controls. 1A: Unadjusted cortical BMD values by age with fitted linear regression lines for HBM cases (black circles) (standardized β = − 0.009 [95%CI (shaded) − 0.021, 0.003], *p* = 0.143) and family controls (FC) (grey triangles) − 0.041 [− 0.059, − 0.023], *p* < 0.001. 1B: Fully adjusted (gender, weight and height, alcohol consumption, smoking status, malignancy and steroid use, and menopausal status and estrogen replacement use in women) regression for cortical BMD by age in HBM cases (− 0.007 [− 0.022, 0.009], *p* = 0.405) and family controls (FC) (− 0.046 [− 0.067, − 0.026), *p* < 0.001), interaction *p* = 0.002. 2A: Unadjusted trabecular BMD values by age with fitted linear regression lines for HBM cases (0.004 [− 0.008, 0.016], *p* = 0.532) and FC (− 0.029 [− 0.042, − 0.016], *p* < 0.001). 2B: Fully adjusted (as above) regression for trabecular BMD by age in HBM cases (− 0.006 [− 0.021, 0.008], *p* = 0.407) and family controls (FC) (− 0.035 [− 0.049, − 0.020), *p* < 0.001), interaction *p* = 0.001. 3A: Unadjusted cortical BMD values by age with fitted linear regression lines for HBM cases (− 0.003 [− 0.017, 0.011], *p* = 0.663) and FC (− 0.014 [− 0.028, 0.000], *p* = 0.050). 3B: Fully adjusted (as above) for cortical BMD by age in HBM cases (− 0.027 [− 0.046, − 0.009], *p* = 0.004) and FC (− 0.025 [− 0.047, − 0.003], *p* = 0.023), interaction *p* = 0.153. 4A: Unadjusted trabecular BMD values by age with fitted linear regression lines for HBM cases (− 0.018 [− 0.030, − 0.007], *p* = 0.002) and FC (− 0.036 [− 0.053, − 0.0182], *p* < 0.001). 4B: Fully adjusted (as above) regression for trabecular BMD by age in HBM cases (− 0.021 [− 0.041, 0.000], *p* = 0.047) and FC (− 0.023 [− 0.044, − 0.003], *p* = 0.027), interaction *p* = 0.424.

The fully adjusted model was used to investigate the strength of associations between age and pQCT parameters of interest, separately in HBM cases and family controls (population controls were omitted as their age range was too narrow). A strong inverse association was seen between age and cBMD at the mid-tibia amongst family controls (adjusted β − 0.046 [− 0.026, − 0.067], *p* < 0.001), but not amongst HBM cases (− 0.007 [− 0.022, 0.009], *p* = 0.405), interaction *p* = 0.002 (Fig. 2, Table 5). In contrast, distal cortical thickness declined with age in a similar pattern in HBM cases and controls. At the distal tibia a strong inverse association was also seen between age and tBMD amongst family controls (adjusted β − 0.035 [− 0.020, − 0.049], *p* < 0.001), but not amongst HBM cases (− 0.006 [− 0.021, 0.008], *p* = 0.407), interaction *p* = 0.001; changes in mid-tibia CBA and SSI followed very similar patterns. TBA increased with age in both HBM cases and controls at the distal tibia.

#### Relationships between pQCT parameters and age: Radius

In contrast to the tibia, cBMD at the mid-radius declined with age in both HBM cases (adjusted β − 0.027 [− 0.009, − 0.046], *p* = 0.004), and family controls (β − 0.025 [− 0.003, − 0.047], *p* = 0.023), without evidence of interaction (*p* = 0.153) (Fig. 2, Table 5). Similar declines in both HBM cases and controls were seen for the proportion of TBA which constituted cortex at the mid-radius, although cortical thickness measured at both the mid and distal radius did not follow such a clear pattern. Further declines with age were seen for radius tBMD in HBM cases (adjusted β − 0.021 [0.000, − 0.041], *p* = 0.047), and family controls (β − 0.023 [− 0.030, − 0.044], *p* = 0.027), (interaction *p* = 0.424). Whilst TBA increased with age at both the mid and distal radius, in both HBM cases and family controls (Table 5).

## Discussion

**Fig. 3:**
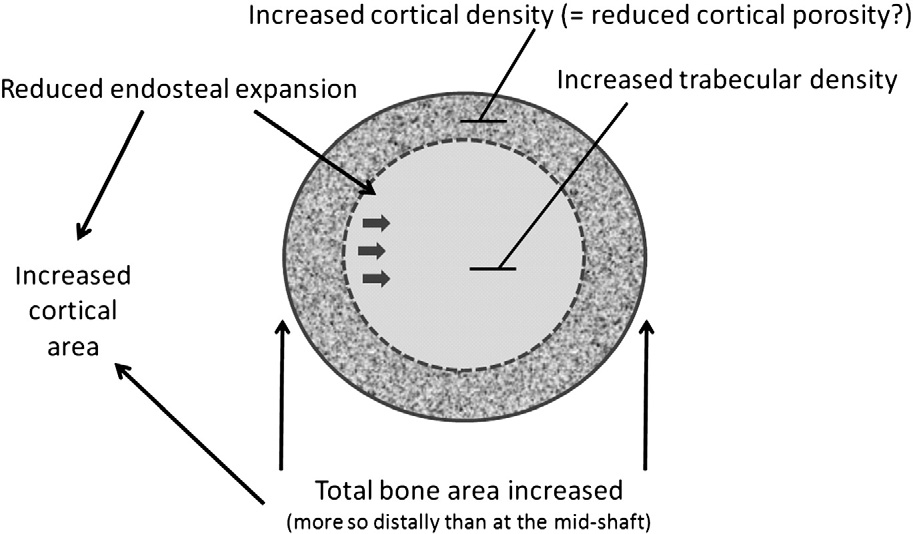
Theoretical representation of changes observed in long bones of HBM individuals based upon distal and mid-tibia pQCT findings.

This study is the first to use pQCT to define the bone phenotype of a large population of individuals with unexplained HBM. We found HBM cases, identified by screening routine NHS DXA scans, to have both a characteristic cortical and trabecular phenotype (Fig. 3). In terms of the former, after taking into account confounding factors, HBM was characterised by increased cBMD, thicker cortices, and larger TBA which was most apparent distally. The net effect of these differences produced an increase in CBA, and in estimated cortical bone strength as reflected by SSI. In terms of the trabecular phenotype, trabecular density was markedly increased in HBM. These phenotypes affected men and women equally.

The increase in TBA in HBM cases was most marked distally (approximately 20% greater than controls) and was only apparent at the mid-shaft of both tibia and radius after adjustment for confounding factors (approximately 4% greater). Increased TBA may reflect enhanced periosteal apposition secondary to increased osteoblast activity. However, the greater proportion of cortical bone within the tibia and radius of HBM bones would also support reduced endosteal expansion. Any tendency for reduced bone turnover in HBM cases is likely to have contributed to the observed higher cBMD, by reducing cortical porosity, and prolonging the time available for secondary mineralisation. Unfortunately we were unable to explore this aspect of the phenotype in more detail, since bone biopsies were not performed. TBA tended to increase with age to a similar extent in controls and HBM cases, particularly at the radius, suggesting the greater TBA in HBM largely arises in earlier life prior to accrual of peak bone mass.

At the tibia, the differences in tBMD and cBMD between HBM cases and family controls increased substantially with age, reflecting a decrease in these parameters in controls which was not seen in HBM cases. Since age-related decreases in trabecular and cortical density are likely to be mediated by increased bone resorption, absence of these age-related changes in HBM cases may reflect some form of protection against excessive osteoclast activity. In contrast, although radial tBMD and cBMD were greater in HBM cases for any given age, these parameters declined with age to the same extent in both HBM cases and controls, suggesting there may be an interaction between age-related changes in cortical and trabecular BMD, HBM case status and weight-bearing activity.

Our results suggest the HBM phenotype might arise through a combination of excessive osteoblast activity and reduced osteoclast activity. This raises the possibility of two distinct biological actions on bone. The genetic basis remains unknown, and could theoretically arise from a single gene mutation with pleiotropic effects, or from multiple variants with diverse effects. Phenotypic analysis of HBM families arising from an activating *LRP5* mutation revealed a similar phenotype to that observed here, with higher total cortical areas suggestive of increased periosteal apposition, but also increased cBMD, increased cortical thickness and reduced bone turnover indicative of reduced bone resorption [3]. Rather than reflecting two distinct biological effects, recent animal studies suggest that *LRP5* activation leads to increased mechanosensory responsiveness, resulting in a cortical bone phenotype similar to that reported here, characterised by a combination of increased osteoblast and reduced osteoclast activities [15]. Our observation that age-associated declines in cortical and trabecular BMD appeared attenuated in the lower rather than upper limb is consistent with increased responsiveness to mechanical strain possibly contributing to the HBM skeletal phenotype. In fact, direct sequencing of our 98 HBM cases for mutations affecting exons 2, 3 and 4 of *LRP5* and the entire coding region of *SOST* have thus far identified causative mutations in only one individual [16], whose pQCT parameters lay within the HBM distribution as a whole. Therefore, although enhanced mechanosensory responsiveness may contribute to the cortical bone phenotype observed, this is not generally explained by activating mutations in *LRP5*. The genetic basis underlying currently unexplained HBM will be the focus of future studies.

In several instances, the bone phenotype of family controls was intermediate between that of HBM cases and population controls. Comparisons were made between HBM cases and a second general population-based control group firstly due to concerns that family controls may have limited validity due to shared environmental and heritable factors, and secondly to place HBM results within the context of a general UK population. A clustered analysis was used to allow for within-family clustering of shared factors. Although the effect of unmeasured environmental factors such as strontium in soil cannot be excluded, BMD Z-scores >+ 3 are unlikely to be explained by such factors. Our population controls have previously been shown to be representative of the UK population in terms of BMD and smoking habits, improving generalizability [10]. Baseline differences between HBM cases and family controls reflect our study design given the biases inherent to those referred to NHS DXA services e.g. those receiving steroids, estrogen replacement, or aromatase inhibitors for breast cancer are more likely to be referred for DXA assessment. The 71 index cases (of 98 HBM cases) were more often female so partner controls were more often male [1].

Mid-tibial SSI was substantially greater in HBM cases than controls, suggesting greater bone strength and reduced fracture risk. Application of failure loads to cadaveric specimens has demonstrated a strong association between pQCT measured bone geometric parameters at the radius and fracture points [17–19]. SSI particularly strongly correlates with load to fracture [19]. However, no clear association in overall fracture prevalence has previously been observed in our HBM population [1], although lower- and upper-limb fractures were not differentiated. Longitudinal follow-up of HBM is required to assess fracture incidence.

Our study design has limitations. Our data are not longitudinal and therefore we cannot determine the true age-related changes in bone geometry. Observed associations between HBM cases and population controls may in part be explained by residual confounding as clinical co-variables were collected using different methods; face-to-face interview and self-completed questionnaire respectively. However, differences in the year of data collection, of on average 5 years, are unlikely to have introduced any significant confounding by period effect and family controls were assessed contemporaneously. Hull, in the North of England where HBM cases and family controls were recruited, and Hertfordshire, in the South from where our population controls originated, may well differ in terms of lifestyle, socio-economic position and medical practice. For example, a greater proportion of HBM cases had a history of estrogen replacement use, than had population controls, which may reflect historical regional prescribing preferences [20,21]. Physical activity data were available for HBM cases and family controls, but not population controls. Whilst further adjustment made no material difference to family-based analyses, residual confounding by physical activity cannot be excluded from population control analyses. In addition, sample size restricted our ability to determined gender-specific age-associated changes in HBM bone geometry, as previously identified within the general population [22].

pQCT has some inherent technical limitations. Non-differential partial volume effect (PVE) may bias pQCT parameter differences between HBM cases and controls, as PVE has a greater impact on thinner than thicker cortices. Furthermore, a larger tibia will be less prone to PVE than a smaller radius, possibly explaining some of the weaker trends observed in the upper limb, although application of a PVE correction algorithm did not materially influence results [23]. We used a standard voxel size of 0.5 mm (resolution 500 μm) which is both time efficient and avoids areal measurement drift of cortical densities [24]. Cortical thickness is often not measurable at the 4% level of the distal tibia/radius, as cortical thinning leads to inconsistencies in the cortical shell contour, although the cortex was clearly visible on visual inspection of HBM pQCT images. However, with resolution 500 μm, small changes in cortical bone loss may be missed. Moreover, differences in age-related changes in trabecular BMD might reflect an artefact secondary to trabecularisation of the cortex, given the greater cortical thickness in HBM cases. Comparisons with other published values for pQCT measured bone parameters are problematic as methods, scan sites and threshold settings vary greatly. No consensus regarding optimal pQCT methodology currently exists and reference data are limited; pQCT density measurements from different devices cannot be compared [25].

### Conclusion

We used pQCT to study the skeletal phenotype of HBM cases identified by screening NHS DXA databases, comparing our results with both family and population-based controls. As well as alterations in trabecular bone, comprising increased trabecular BMD, HBM cases showed a marked cortical bone phenotype, comprising increased cBMD, TBA, CBA and cortical thickness (Fig. 3). An increase in predicted cortical bone strength was also observed as reflected by SSI. Further analysis suggested HBM cases may experience attenuated age-related declines in tBMD, cBMD, CBA and SSI in weight bearing but not non-weight bearing bones, possibly suggesting resistance to higher rates of bone remodelling associated with ageing, potentially reflecting altered mechanosensitivity. Future studies are justified to understand the basis for this phenotype, for example by investigating its genetic origins, as a means of defining new pathways involved in the pathogenesis of age-related bone loss.

## Figures and Tables

**Table 1:** Clinical characteristics of high bone mass cases, compared firstly with family controls and secondly with general population controls.

	HBM cases (*n* = 98)	Family controls (*n* = 63)			General population controls (*n* = 691)		
	Mean (SD)	Mean (SD)	Mean difference (95%CI)	*p* value	Mean (SD)	Mean difference (95%CI)	*p* value
Age (years)	61.0 (13.8)	55.5 (15.8)	5.5 (1.0, 10.0)	0.017	69.3 (2.6)	− 8.5 (− 9.7, − 7.3)	< 0.001
Height (cm)	166.8 (8.0)	171.0 (10.0)	− 4.2 (− 6.9,,−1.5)	0.002	167.0 (9.2)	− 0.02 (− 2.0, 1.9)	0.982
Weight (kg)	86.4 (16.7)	82.3 (16.9)	4.1 (− 1.3, 9.5)	0.132	75.8 (16.7)	10.7 (7.8, 13.6)	< 0.001
BMI (kg/m^2^)	31.1 (6.0)	28.1 (5.0)	3.0 (1.4, 4.6)	< 0.001	27.1 (6.0)	4.0 (3.0, 4.9)	< 0.001
L1 Z-score	3.7 (1.1)^d^	0.4 (1.3)	3.26 (2.89, 3.63)		0.9 (1.4)	2.77 (2.47, 3.08)	
Total hip Z-score^c^	3.0 (1.0)^d^	0.4 (0.8)	2.54 (2.25, 2.84)		0.8 (1.0)	2.15 (1.94, 2.37)	

	*n* (%)	*n* (%)	OR (95%CI)	*p* value	*n* (%)	OR (95%CI)	*p* value

Female	80 (81.6)	33 (50.8)	4.31 (2.13, 8.73)	< 0.001	299 (50.3)	4.38 (2.57, 7.49)	< 0.001
Post-menopausal	65 (81.3)	17 (51.5)	5.35 (5.35, 1.73)	0.004	287 (99.0)	0.05 (0.01, 0.16)	< 0.001
Estrogen replacement^a^	44 (58.7)	8 (27.6)	7.84 (7.84, 1.63)	0.010	121 (20.8)	5.40 (3.27, 8.91)	< 0.001
Malignancy^a^	14 (14.3)	4 (6.2)	2.54 (2.54, 0.80)	0.115	25 (4.3)	3.71 (1.85, 7.41)	< 0.001
Steroid use^a^	28 (28.6)	13 (20.0)	1.60 (1.60, 0.76)	0.219	9 (1.5)	25.4 (11.5, 56.1)	< 0.001
Fracture since aged 45	7(8.3)^b^	3(6.7) ^b^	1.27 (0.31, 5.18)	0.736	111 (19.4)	0.38 (0.17, 0.84)	0.017
Self-reported alcohol consumption							
None	27 (27.6)	14 (21.5)	1.00		84 (14.5)	1.00	
Occasional	13 (13.3)	5 (7.7)	1.36 (1.36, 0.40)	0.026	107 (18.5)	0.38 (0.18, 0.78)	0.019
Regular	51 (52.0)	29 (44.6)	0.92 (0.92, 0.41)		331 (57.2)	0.48 (0.28, 0.81)	
Heavy	7 (7.1)	17 (26.2)	0.21 (0.21, 0.07)		57 (9.8)	0.38 (0.16, 0.94)	
Self-reported smoking status							
Never	38 (38.8)	27 (41.5)	1.00		298 (51.5)	1.00	
Ex-smoker	45 (45.9)	29 (44.6)	1.10 (1.10, 0.56)	0.930	241 (41.6)	1.46 (0.92, 2.33)	0.008
Current	15 (15.3)	9 (13.8)	1.18 (1.18, 0.45)		39 (6.7)	3.02 (1.52, 5.98)	

Continuous and categorical data presented (without adjustment). BMI: body mass index, CI: confidence interval, OR: odds ratio, SD: standard deviation, L1: 1st lumbar vertebra. ^a^Ever reported. ^b^Limited to participants aged > 45 years. ^c^Maximum of left and right total hip Z-scores. ^d^Amongst HBM cases 36 had a L1 Z-score ≥+ 3.2 without a total hip Z-score ≥+ 3.2 and 12 had a total hip Z-score ≥+ 3.2 without a L1 Z-score ≥+ 3.2.

**Table 2:** Unadjusted distal and mid-shaft tibial pQCT measures in high bone mass cases compared with firstly family controls and secondly population controls.

		HBM cases (*n* = 98)	Family controls (*n* = 63)			General population controls (*n* = 691)		
Site		Mean (95%CI)	Mean (95%CI)	Mean difference (95%CI)	*p* value	Mean (95%CI)	Mean difference (95%CI)	*p* value
4% distal tibia	Total BA (mm^2^)	1102 (1059, 1146)	996 (941, 1051)	107 (38.0, 175)	0.002	965 (945, 985)	140 (86.4, 194)	< 0.001
	Trabecular BMD (mg/cm^3^)	315 (308, 322)	278 (269, 287)	37.4 (26.2, 48.7)	< 0.001	270 (267, 273)	45.1 (36.5, 53.6)	< 0.001
	Cortical thickness (mm)	1.26 (1.08, 1.44)	1.12 (0.89, 1.34)	0.14 (− 0.15, 0.43)	0.344	0.53 (0.47, 0.58)	0.73 (0.59, 0.88)	< 0.001
66% mid-shaft tibia	Total BA (mm^2^)	630 (609, 651)	653 (628, 679)	− 23.0 (− 56.0, 10.1)	0.173	602 (593, 611)	29.6 (6.6, 52.7)	0.012
	Cortical BMD (mg/cm^3^)	1128 (1119, 1136)	1111 (1101, 1122)	16.3 (2.9, 29.7)	0.017	1078 (1075, 1081)	49.7 (40.9, 58.6)	< 0.001
	Cortical thickness (mm)	4.54 (4.39, 4.69)	4.23 (4.04, 4.42)	0.31 (0.08, 0.55)	0.010	4.36 (4.30, 4.43)	0.18 (0.00, 0.35)	0.044
	Cortical BA (mm^2^)	337 (325, 350)	325 (310, 340)	12.0 (− 7.3, 31.3)	0.223	315 (310, 321)	22.0 (6.7, 37.3)	0.005
	Cortical/total BA (%)	54.0 (52.4, 55.6)	50.1 (48.1, 52.1)	3.9 (1.6, 6.3)	0.001	53.8 (53.2, 54.5)	0.03 (− 1.7, 1.8)	0.969
	SSI (mm^3^)	1643 (1563, 1723)	1636 (1540, 1733)	6.6 (− 119, 132)	0.918	1441 (1407, 1475)	204 (112, 296)	< 0.001

HBM: high bone mass, BA: bone area, BMD: bone mineral density, CI: confidence interval, SSI: strength strain index.

**Table 3:** Fully adjusted distal and mid-shaft tibial pQCT measures in High Bone Mass cases compared with firstly family controls and secondly population controls.

		HBM cases (*n* = 98)	Family controls (*n* = 63)			General population controls (*n* = 691)		
Site		Mean (95%CI)	Mean (95%CI)	Mean difference (95%CI)	*p* value	Mean (95%CI)	Mean difference (95%CI)	*p* value
4% distal tibia	Total BA (mm^2^)	1239 (1107, 1370)	1037 (915, 1160)	201 (146, 257)	< 0.001	1097 (1052, 1141)	212 (167, 256)	< 0.001
	Trabecular BMD (mg/cm^3^)	340 (321, 360)	294 (276, 312)	46.0 (33.5, 58.4)	< 0.001	291 (282, 300)	56.6 (47.7, 65.5)	< 0.001
	Cortical thickness (mm)	2.10 (1.40, 2.81)	1.54 (0.88, 2.20)	0.57 (0.27, 0.87)	< 0.001	0.90 (0.75, 1.04)	1.01 (0.86, 1.15)	< 0.001
66% mid-shaft tibia	Total BA (mm^2^)	668 (608, 728)	642 (586, 698)	25.4 (0.1, 50.6)	0.049	636 (619, 653)	52.3 (35.5, 69.2)	< 0.001
	Cortical BMD (mg/cm^3^)	1130 (1094, 1166)	1101 (1068, 1135)	28.5 (13.4, 43.7)	< 0.001	1093 (1083, 1103)	58.0 (48.0, 68.0)	< 0.001
	Cortical thickness (mm)	5.32 (4.77, 5.86)	4.73 (4.22, 5.23)	0.59 (0.36, 0.81)	< 0.001	4.82 (4.65, 5.00)	0.47 (0.30, 0.64)	< 0.001
	Cortical BA (mm^2^)	401 (370, 432)	357 (327, 386)	44.5 (31.6, 57.4)	< 0.001	354 (341, 367)	47.7 (34.4, 61.0)	< 0.001
	Cortical/total BA (%)	59.9 (53.7, 66.2)	54.8 (49.0, 60.6)	5.2 (2.5, 7.8)	< 0.001	56.7 (54.7, 58.6)	1.9 (− 0.1, 3.9)	0.064
	SSI (mm^3^)	1974 (1789, 2159)	1720 (1548, 1891)	254 (176, 332)	< 0.001	1654 (1579, 1729)	346 (272, 421)	< 0.001

HBM: high bone mass, BA: bone area, BMD: bone mineral density, CI: confidence interval, SSI: strength strain index. Adjusted for age, weight and height, alcohol consumption, smoking status, malignancy and steroid use, and menopausal status and estrogen replacement use in women.

**Table 4:** Gender-stratified fully adjusted distal and mid-shaft tibial pQCT measures in high bone mass cases compared with firstly family controls and secondly population controls.

		Mean (95%CI)	Mean (95%CI)	Mean difference (95%CI)	*p* value	Mean (95%CI)	Mean difference (95%CI)	*p* value
Female		HBM cases (*n* = 80)	Family controls (*n* = 32)			General population controls (*n* = 299)		
4% distal tibia	Total BA (mm^2^)	1063 (955, 1172)	836 (728, 945)	227 (155, 299)	< 0.001	811 (763, 859)	213 (160, 266)	< 0.001
	Trabecular BMD (mg/cm^3^)	295 (270, 320)	250 (225, 275)	45.1 (28.4, 61.7)	0.010	250 (240, 260)	54.4 (43.2, 65.7)	< 0.001
	Cortical thickness (mm)	1.19 (0.64, 1.74)	0.73 (0.18, 1.29)	0.46 (0.09, 0.82)	0.015	0.19 (0.09, 0.30)	0.92 (0.80, 1.03)	< 0.001
66% mid-shaft tibia	Total BA (mm^2^)	599 (547, 650)	576 (526, 627)	22.5 (− 11.3, 56.3)	0.192	564 (548, 581)	48.6 (30.3, 67.0)	< 0.001
	Cortical BMD (mg/cm^3^)	1119 (1086, 1152)	1096 (1063, 1128)	23.7 (2.4, 45.0)	0.029	1066 (1055, 1078)	60.0 (47.1, 72.9)	< 0.001
	Cortical thickness (mm)	4.36 (3.90, 4.81)	3.87 (3.42, 4.32)	0.48 (0.19, 0.78)	0.001	4.02 (3.86, 4.18)	0.41 (0.23, 0.60)	< 0.001
	Cortical BA (mm^2^)	320 (295, 344)	281 (257, 305)	38.9 (22.8, 54.9)	< 0.001	286 (277, 295)	40.9 (30.8, 60.0)	< 0.001
	Cortical/total BA (%)	52.4 (46.9, 58.0)	48.2 (42.7, 53.7)	4.2 (0.6, 7.9)	0.023	50.6 (48.5, 52.6)	1.8 (− 0.6, 4.1)	0.136
	SSI (mm^3^)	1532 (1385, 1679)	1324 (1180, 1469)	208 (112, 305)	< 0.001	1269 (1223, 1315)	301 (249, 352)	< 0.001

Male		HBM cases (*n* = 18)	Family controls (*n* = 31)			General population controls (*n* = 295)		

4% distal tibia	Total BA (mm^2^)	1233 (1022, 1445)	1098 (898, 1298)	135 (32.3, 238)	0.010	1145 (1057, 1233)	199 (107, 292)	< 0.001
	Trabecular BMD (mg/cm^3^)	327 (287, 366)	281 (244, 318)	45.6 (25.2, 66.0)	< 0.001	295 (279, 311)	63.2 (46.1, 80.3)	< 0.001
	Cortical thickness (mm)	1.6 (0.6, 2.6)	0.94 (− 0.03, 1.90)	0.66 (0.08, 1.24)	0.025	0.84 (0.48, 1.21)	1.17 (0.79, 1.56)	< 0.001
66% mid-shaft tibia	Total BA (mm^2^)	742 (676, 809)	706 (640, 771)	36.6 (− 3.3, 76.4)	0.073	606 (570, 643)	76.2 (37.9, 115)	< 0.001
	Cortical BMD (mg/cm^3^)	1108 (1076, 1140)	1085 (1054, 1116)	23.0 (5.0, 41.0)	0.012	1103 (1086, 1120)	43.3 (25.1, 61.4)	< 0.001
	Cortical thickness (mm)	5.62 (4.87, 6.37)	5.00 (4.27, 5.73)	0.62 (0.21, 1.02)	0.003	5.14 (4.78, 5.51)	0.46 (0.07, 0.84)	0.020
	Cortical BA (mm^2^)	437 (3889, 485)	382 (335, 429)	55.1 (28.4, 81.8)	< 0.001	373 (338, 408)	61.1 (24.3, 97.9)	0.001
	Cortical/total BA (%)	60.2 (52.8, 67.6)	55.7 (48.5, 62.9)	4.5 (0.5, 8.5)	0.028	59.1 (55.1, 63.1)	0.6 (− 3.7, 4.8)	0.797
	SSI (mm^3^)	2199 (1935, 2463)	1857 (1597, 2116)	343 (185, 500)	< 0.001	1688 (1485, 1891)	490 (275, 704)	< 0.001

HBM: high bone mass, BA: bone area, BMD: bone mineral density, CI: confidence interval, SSI: strength strain index. Adjusted for age, weight and height, alcohol consumption, smoking status, malignancy and steroid use (and menopausal status and estrogen replacement use in women).

**Table 5:** Fully adjusted regression coefficients for changes in tibia and radius pQCT parameters with age in HBM cases and family controls.

			*n*	Adjusted β (95% CI)	*p* value	Int. p^a^
4% distal tibia	Total BA	HBM cases	96	0.016 (0.004, 0.028)	0.010	0.413
	(mm^2^)	Family controls	63	0.023 (0.011, 0.034)	< 0.001	
	Trabecular BMD	HBM cases	96	− 0.006 (− 0.021, 0.008)	0.407	0.001
	(mg/cm^3^)	Family controls	63	− 0.035 (− 0.049, − 0.020)	< 0.001	
	Cortical thickness	HBM cases	96	− 0.019 (− 0.036, − 0.001)	0.035	0.358
	(mm)	Family controls	63	− 0.023 (− 0.039, − 0.007)	0.005	
66% mid-shaft tibia	Total BA	HBM cases	91	0.013 (0.000, 0.026)	0.058	0.293
	(mm^2^)	Family controls	64	0.005 (− 0.007, 0.016)	0.404	
	Cortical BMD	HBM cases	91	− 0.007 (− 0.022, 0.009)	0.405	0.002
	(mg/cm^3^)	Family controls	64	− 0.046 (− 0.067, − 0.026)	< 0.001	
	Cortical thickness	HBM cases	88	0.011 (− 0.003, 0.026)	0.128	0.542
	(mm)	Family controls	61	0.000 (− 0.012, 0.013)	0.943	
	Cortical BA	HBM cases	91	0.003 (− 0.008, 0.015)	0.540	0.009
	(mm^2^)	Family controls	64	− 0.016 (− 0.027, − 0.004)	0.007	
	Cortical/total BA	HBM cases	91	− 0.010 (− 0.028, 0.007)	0.237	0.264
	(%)	Family controls	64	− 0.028 (− 0.046, − 0.010)	0.002	
	SSI	HBM cases	91	0.006 (− 0.004, 0.016)	0.249	0.005
	(mm^3^)	Family controls	64	− 0.011 (− 0.020, − 0.001)	0.034	
4% distal radius	Total BA	HBM cases	95	0.019 (0.003, 0.035)	0.022	0.706
	(mm^2^)	Family controls	65	0.016 (− 0.002, 0.035)	0.087	
	Trabecular BMD	HBM cases	95	− 0.021 (− 0.041, 0.000)	0.047	0.424
	(mg/cm^3^)	Family controls	65	− 0.023 (− 0.044, − 0.003)	0.027	
	Cortical thickness	HBM cases	95	0.010 (− 0.005, 0.025)	0.204	0.835
	(mm)	Family controls	65	0.001 (− 0.033, 0.035)	0.950	
60% mid-shaft radius	Total BA	HBM cases	94	0.025 (0.008, 0.041)	0.003	0.915
	(mm^2^)	Family controls	63	0.015 (0.000, 0.030)	0.051	
	Cortical BMD	HBM cases	94	− 0.027 (− 0.046, − 0.009)	0.004	0.153
	(mg/cm^3^)	Family controls	63	− 0.025 (− 0.047, − 0.003)	0.023	
	Cortical thickness	HBM cases	94	− 0.010 (− 0.027, 0.006)	0.227	0.353
	(mm)	Family controls	63	− 0.029 (− 0.049, − 0.008)	0.006	
	Cortical BA	HBM cases	94	0.006 (− 0.009, 0.022)	0.411	0.538
	(mm^2^)	Family controls	63	− 0.009 (− 0.024, 0.007)	0.267	
	Cortical/total BA	HBM cases	94	− 0.024 (− 0.042, − 0.006)	0.011	0.219
	(%)	Family controls	63	− 0.041 (− 0.063, − 0.018)	< 0.001	
	SSI	HBM cases	94	0.013 (− 0.003, 0.029)	0.103	0.597
	(mm^3^)	Family controls	63	0.001 (− 0.014, 0.016)	0.886	

β represents number of SD changes in each pQCT parameter per year increase in age. ^a^*p* value for interaction. Adjusted for weight and height, alcohol consumption, smoking status, malignancy and steroid use, and menopausal status and estrogen replacement use in women.
