# Supplementary material for: The high bone mass phenotype is characterised by a combined cortical and trabecular bone phenotype: Findings from a pQCT case–control study
Source: Bone. 2013 Jan;52(1):380–8. doi: 10.1016/j.bone.2012.10.021 (PMC3526774; doi:10.1016/j.bone.2012.10.021)
Supplement: Supplementary Tables [file mmc1.docx]

**Supplementary table 1s: Unadjusted distal and mid-shaft radial pQCT measures in High Bone Mass cases compared with firstly family controls and secondly population controls**

|  |  | **HBM cases n=96** | **Family controls n=63** | | | **General population controls n=691** | | |
| --- | --- | --- | --- | --- | --- | --- | --- | --- |
| **Site** |  | **Mean (95%CI)** | **Mean (95%CI)** | **Mean difference (95%CI)** | **p value** | **Mean (95%CI)** | **Mean difference (95%CI)** | **p value** |
| **4% Distal**  **Radius** |  |  |  |  |  |  |  |  |
|  | Total BA (mm^2^) | 321 (304, 338) | 296 (275, 317) | 24.7 (-2.4, 51.8) | 0.074 | 284 (277, 291) | 36.8 (17.1, 56.6) | <0.001 |
|  | Trabecular BMD (mg/cm^3^) | 287 (280, 294) | 264 (256, 273) | 23.3 (12.4, 34.1) | <0.001 | 258 (254, 261) | 28.9 (20.8, 37.0) | <0.001 |
|  | Cortical thickness (mm) | 0.67 (0.61, 0.73) | 0.59 (0.52, 0.65) | 0.08 (0.00, 0.17) | 0.063 | 0.48 (0.46, 0.51) | 0.19 (0.12, 0.25) | <0.001 |
|  |  |  |  |  |  |  |  |  |
| **60% Mid-shaft**  **Radius** | Total BA (mm^2^) | 159 (153, 165) | 162 (154, 169) | -2.4 (-11.4, 6.6) | 0.605 | Not available |  |  |
|  | Cortical BMD (mg/cm^3^) | 1171 (1162, 1180) | 1157 (1146, 1167) | 14.4 (0.6, 28.1) | 0.040 | Not available |  |  |
|  | Cortical thickness (mm) | 2.78 (2.70, 2.86) | 2.68 (2.58, 2.78) | 0.10 (-0.03, 0.22) | 0.122 | Not available |  |  |
|  | Cortical BA (mm^2^) | 99.6 (95.9, 103) | 97.6 (93.1, 102) | 2.0 (-3.8, 7.7) | 0.496 | Not available |  |  |
|  | Cortical/Total BA (%) | 63.0 (61.6, 64.4) | 60.7 (59.0, 62.4) | 2.2 (0.03, 4.4) | 0.047 | Not available |  |  |
|  | SSI (mm^3^) | 239 (226, 251) | 236 (221, 251) | 2.6 (-17.1, 22.4) | 0.794 | Not available |  |  |

HBM: High Bone Mass, BA: Bone area, BMD: Bone Mineral Density, CI: Confidence Interval, SSI: Strength Strain Index.

pQCT at the 60% site was not performed in population controls

**Supplementary table 2s: Fully-adjusted distal and mid-shaft radial pQCT measures in High Bone Mass cases compared with firstly family controls and secondly population controls**

|  |  | **HBM cases n=96** | **Family controls n=63** | | | **General population controls n=691** | | |
| --- | --- | --- | --- | --- | --- | --- | --- | --- |
| **Site** |  | **Mean (95%CI)** | **Mean (95%CI)** | **Mean difference (95%CI)** | **p value** | **Mean (95%CI)** | **Mean difference (95%CI)** | **p value** |
| **4% Distal**  **Radius** |  |  |  |  |  |  |  |  |
|  | Total BA (mm^2^) | 403 (346, 461) | 339 (286, 393) | 64.0 (40.0, 88.0) | <0.001 | 343 (328, 358) | 72.9 (57.8, 88.0) | <0.001 |
|  | Trabecular BMD (mg/cm^3^) | 316 (296, 336) | 285 (266, 304) | 31.0 (18.1, 43.8) | <0.001 | 279 (271, 288) | 38.4 (29.9, 46.9) | <0.001 |
|  | Cortical thickness (mm) | 0.99 (0.78, 1.21) | 0.81 (0.61, 1.00) | 0.19 (0.10, 0.28) | <0.001 | 0.63 (0.57, 0.70) | 0.30 (0.23, 0.37) | <0.001 |
|  |  |  |  |  |  |  |  |  |
| **60% Mid-shaft**  **Radius** | Total BA (mm^2^) | 198 (180, 216) | 188 (172, 205) | 9.6 (2.2, 16.9) | 0.011 | Not available |  |  |
|  | Cortical BMD (mg/cm^3^) | 1144 (1108, 1180) | 1116 (1083, 1149) | 27.8 (12.9, 42.7) | <0.001 | Not available |  |  |
|  | Cortical thickness (mm) | 2.98 (2.68, 3.28) | 2.67 (2.39, 2.94) | 0.32 (0.19, 0.44) | <0.001 | Not available |  |  |
|  | Cortical BA (mm^2^) | 120 (109, 131) | 107 (96.7, 117) | 13.2 (8.6, 17.7) | <0.001 | Not available |  |  |
|  | Cortical/Total BA (%) | 60.9 (54.9, 66.8) | 56.4 (50.9, 61.8) | 4.5 (2.0, 6.9) | <0.001 | Not available |  |  |
|  | SSI (mm^3^) | 315 (278, 353) | 277 (243, 311) | 38.3 (22.9, 53.7) | <0.001 | Not available |  |  |

HBM: High Bone Mass, BA: Bone area, BMD: Bone Mineral Density, CI: Confidence Interval, SSI: Strength Strain Index.

pQCT at the 60% site was not performed in population controls

Adjusted for age, weight & height, alcohol consumption, smoking status, malignancy and steroid use, and menopausal status & estrogen replacement use in women

**Supplementary table 3s: Gender-stratified unadjusted distal and mid-shaft tibial pQCT measures in High Bone Mass cases compared with firstly family controls and secondly population controls**

|  |  | **Mean (95%CI)** | **Mean (95%CI)** | **Mean difference (95%CI)** | **p value** | **Mean (95%CI)** | **Mean difference (95%CI)** | **p value** |
| --- | --- | --- | --- | --- | --- | --- | --- | --- |
| **FEMALE** | | **HBM cases n=80** | **Family controls n=32** | | | **General population controls n=299** | | |
| **4% Distal**  **Tibia** | Total BA (mm^2^) | 1045 (1009, 1082) | 824 (767, 882) | 221 (158, 285) | <0.001 | 793 (771, 816) | 258 (210, 307) | <0.001 |
|  | Trabecular BMD (mg/cm^3^) | 310 (303, 317) | 262 (250, 274) | 47.8 (33.9, 61.8) | <0.001 | 256 (251, 260) | 54.2 (44.2, 64.3) | <0.001 |
|  | Cortical thickness (mm) | 1.09 (0.91, 1.28) | 0.87 (0.58, 1.16) | 0.22 (-0.12, 0.56) | 0.207 | 0.2 (0.15, 0.25) | 0.9 (0.79, 1.01) | <0.001 |
|  |  |  |  |  |  |  |  |  |
| **66% Mid-shaft**  **Tibia** | Total BA (mm^2^) | 595 (576, 614) | 574 (545, 603) | 20.7 (-9.2, 50.6) | 0.175 | 528 (520, 536) | 76.1 (58.3, 93.8) | <0.001 |
|  | Cortical BMD (mg/cm^3^) | 1127 (1117, 1137) | 1111 (1095, 1127) | 15.9 (-3.3, 35.1) | 0.104 | 1069 (1064, 1074) | 57.7 (46.6, 68.8) | <0.001 |
|  | Cortical thickness (mm) | 4.39 (4.25, 4.54) | 3.80 (3.58, 4.02) | 0.59 (0.33, 0.86) | <0.001 | 3.97 (3.89, 4.04) | 0.42 (0.25, 0.58) | <0.001 |
|  | Cortical BA (mm^2^) | 318 (310, 327) | 274 (261, 287) | 44.1 (28.3, 60.0) | <0.001 | 271 (266, 275) | 47.9 (37.7, 58.1) | <0.001 |
|  | Cortical/Total BA (%) | 53.5 (51.8, 55.3) | 48.3 (45.7, 51.0) | 5.2 (2.0, 8.4) | 0.001 | 52.0 (51.1, 52.9) | 1.4 (-0.6, 3.4) | 0.172 |
|  | SSI (mm^3^) | 1505 (1450, 1560) | 1302 (1216, 1388) | 203 (106, 301) | <0.001 | 1154 (1129, 1178) | 364 (310, 419) | <0.001 |
| **MALE** | | **HBM cases n=18** | **Family controls n=31** | | | **General population controls n=295** | | |
| **4% Distal**  **Tibia** | Total BA (mm^2^) | 1233 (1022, 1445) | 1098 (898, 1298) | 135 (32.3, 238) | 0.010 | 1145 (1057, 1233) | 199 (107, 292) | <0.001 |
|  | Trabecular BMD (mg/cm^3^) | 335 (320, 350) | 293 (281, 305) | 41.9 (24.9, 58.9) | <0.001 | 285 (281, 289) | 54.0 (38.4, 69.7) | <0.001 |
|  | Cortical thickness (mm) | 1.6 (0.6, 2.6) | 0.94 (-0.03, 1.9) | 0.66 (0.08, 1.24) | 0.025 | 0.84 (0.48, 1.21) | 1.17 (0.79, 1.56) | <0.001 |
|  |  |  |  |  |  |  |  |  |
| **66% Mid-shaft**  **Tibia** | Total BA (mm^2^) | 742 (676, 809) | 706 (640, 771) | 36.6 (-3.3, 76.4) | 0.073 | 606 (570, 643) | 76.2 (37.9, 114) | <0.001 |
|  | Cortical BMD (mg/cm^3^) | 1108 (1076, 1140) | 1085 (1054, 1116) | 23.0 (5.0, 41.0) | 0.012 | 1103 (1086, 1120) | 43.3 (25.1, 61.4) | <0.001 |
|  | Cortical thickness (mm) | 5.62 (4.87, 6.37) | 5.0 (4.27, 5.73) | 0.62 (0.21, 1.02) | 0.003 | 5.14 (4.78, 5.51) | 0.46 (0.07, 0.84) | 0.020 |
|  | Cortical BA (mm^2^) | 437 (389, 485) | 382 (335, 429) | 55.1 (28.4, 81.8) | <0.001 | 373 (338, 408) | 61.1 (24.3, 97.9) | 0.001 |
|  | Cortical/Total BA (%) | 60.2 (52.8, 67.6) | 55.7 (48.5, 62.9) | 4.5 (0.5, 8.5) | 0.028 | 59.1 (55.1, 63.1) | 0.6 (-3.7, 4.8) | 0.797 |
|  | SSI (mm^3^) | 2199 (1935, 2463) | 1857 (1597, 2116) | 343 (185, 500) | <0.001 | 1688 (1485, 1891) | 490 (275, 704) | <0.001 |

HBM: High Bone Mass, BA: Bone area, BMD: Bone Mineral Density, CI: Confidence Interval, SSI: Strength Strain Index
